# First non-invasive magnetic phrenic nerve and diaphragm stimulation in anaesthetized patients: a proof-of-concept study

**DOI:** 10.1186/s40635-023-00506-6

**Published:** 2023-04-21

**Authors:** Alessandro Panelli, Hermann Georges Bartels, Sven Krause, Michael André Verfuß, Aline Michèle Grimm, Niklas Martin Carbon, Julius J. Grunow, Diego Stutzer, Thomas Niederhauser, Laurent Brochard, Steffen Weber-Carstens, Stefan J. Schaller

**Affiliations:** 1 Department of Anesthesiology and Operative Intensive Care Medicine (CVK, CCM), Charité - Universitätsmedizin Berlin, corporate member of Freie Universität Berlin and Humboldt-Universität Zu Berlin, Charitéplatz 1, 10117 Berlin, Germany; 2 Institute for Human Centered Engineering, Bern University of Applied Sciences, Biel, Switzerland; 3 Keenan Research Centre for Biomedical Science, Li Ka Shing Knowledge Institute, Unity Health Toronto, Toronto, ON Canada; 4 Interdepartmental Division of Critical Care, University of Toronto, Toronto, Canada; 5 Department of Anesthesiology and Intensive Care, School of Medicine, Klinikum Rechts Der Isar, Technical University of Munich, Munich, Germany

**Keywords:** Respiration, Artificial, Mechanical ventilation weaning, Muscle weakness, Phrenic nerve, Magnetic field therapy

## Abstract

**Background:**

Mechanical ventilation has side effects such as ventilator-induced diaphragm dysfunction, resulting in prolonged intensive care unit length of stays. Artificially evoked diaphragmatic muscle contraction may potentially maintain diaphragmatic muscle function and thereby ameliorate or counteract ventilator-induced diaphragm dysfunction. We hypothesized that bilateral non-invasive electromagnetic phrenic nerve stimulation (NEPNS) results in adequate diaphragm contractions and consecutively in effective tidal volumes.

**Results:**

This single-centre proof-of-concept study was performed in five patients who were 30 [IQR 21–33] years old, 60% (*n* = 3) females and undergoing elective surgery with general anaesthesia. Following anaesthesia and reversal of muscle relaxation, patients received bilateral NEPNS with different magnetic field intensities (10%, 20%, 30%, 40%); the stimulation was performed bilaterally with dual coils (connected to one standard clinical magnetic stimulator), specifically designed for bilateral non-invasive electromagnetic nerve stimulation. The stimulator with a maximal output of 2400 Volt, 160 Joule, pulse length 160 µs at 100% intensity was limited to 50% intensity, i.e. each single coil had a maximal output of 0.55 Tesla and 1200 Volt. There was a linear relationship between dosage (magnetic field intensity) and effect (tidal volume, primary endpoint, *p* < 0.001). Mean tidal volume was 0.00, 1.81 ± 0.99, 4.55 ± 2.23 and 7.43 ± 3.06 ml/kg ideal body weight applying 10%, 20%, 30% and 40% stimulation intensity, respectively. Mean time to find an initial adequate stimulation point was 89 (range 15–441) seconds.

**Conclusions:**

Bilateral non-invasive electromagnetic phrenic nerve stimulation generated a tidal volume of 3–6 ml/kg ideal body weight due to diaphragmatic contraction in lung-healthy anaesthetized patients. Further perspectives in critically ill patients should include assessment of clinical outcomes to confirm whether diaphragm contraction through non-invasive electromagnetic phrenic nerve stimulation potentially ameliorates or prevents diaphragm atrophy.

**Supplementary Information:**

The online version contains supplementary material available at 10.1186/s40635-023-00506-6.

## Background

Phrenic nerve stimulation may be a preventive or therapeutic option to counteract or minimize the progression of ventilator-induced diaphragm dysfunction (VIDD) during mechanical ventilation (MV). VIDD is a side effect of MV and results in weaning failure, prolonged mechanical ventilation, longer intensive care unit (ICU) and hospital length of stay [1].

VIDD develops rapidly within 18–69 h of invasive mechanical ventilation [2, 3]; the estimated prevalence of VIDD for critically ill patients undergoing prolonged MV times is assessed at 80% [4], leading to weaning failure in approximately 20% of cases [5, 6]. In the aftermath of the several epidemic outbreaks that have occurred in the last 20 years and the global SARS-CoV-2 pandemic with up to 25% of patients requiring MV [7], understanding and counteracting VIDD became a central interest to intensivists. The use of diaphragm stimulation (commonly performed through bilateral phrenic nerve stimulation) may be a preventive or therapeutic option to counteract or minimize the progression of VIDD during MV. The hypothesis (nevertheless yet to be assessed) is that diaphragm contraction by bilateral non-invasive electromagnetic phrenic nerve stimulation (NEPNS) could be a prophylaxis for VIDD, improve outcomes in critically ill patients and counteract the pathological mechanisms leading to diaphragm atrophy during MV. In parallel with the results of peripheral muscles stimulation [8], sufficient diaphragmatic contraction by bilateral NEPNS in anaesthesized patients can in all probability prevent diaphragm atrophy, comparable to the physiological breathing cycles [9, 10]; however, no previous studies have been published to assess minimal or maximal dosage of the magnetic field necessary to achieve a sufficient diaphragm contraction. The published feasibility studies used transcranial magnetic stimulation coils [11, 12]. These studies, however, were performed on non-anaesthetized patients and a volitional component could not be excluded. Second, the used coils were oversized, not designed for the phrenic nerves and two manually synchronized devices had to be used due to the fact that both phrenic nerves run independently.

Since new electromagnetic coils specifically designed for the purpose of bilateral peripheral nerve stimulation were available (using one single stimulator), we hypothesized that it would be feasible to stimulate the phrenic nerves repetitively to generate diaphragm contraction non-invasively. For this proof-of-concept, we assumed an adequate diaphragmatic contraction to prevent or ameliorate VIDD achieved with the surrogate of 3 ml/kg ideal body weight (IBW) based on recent developments in lung protective ventilation in patients with acute respiratory distress syndrome with tidal volume targets of 3 ml/kg PBW termed ultra-lung-protective MV [13]. This study aimed to avoid any potential volitional component, performing the stimulations in patients undergoing general anaesthesia. Other components of feasibility that were evaluated were the time required to find the initial capture point and the stability of the stimulation with increasing distance from the initial capture position — both of which will be critical to the clinical acceptability of the technique.

## Methods

The study was registered with the number NCT04946110. Ethical approval was granted by the ethic committee of Charité — Universitätsmedizin Berlin (EA4/083/21 on July 7th 2021).

### Eligibility criteria

Adult patients (between 18 and 60 years of age) with a low anaesthesia risk score (I or II, according to the American Society of Anesthesiologists — ASA classification), and scheduled for elective surgery at the Department of Otorhinolaryngology, Head and Neck Surgery, Charité — Universitätsmedizin Berlin, were eligible for this proof-of-concept study.

Exclusion criteria were chronic lung diseases (e.g., bronchial asthma, COPD); chronic heart disease; known neurological conditions with motor muscle weakness; known paralysis of the phrenic nerve; inability to read and understand German for the consent; implanted cardiac support systems (e.g., pacemaker, implanted defibrillator); implanted medical pumps; pregnancy.

### Conduct of the study

After preparation of the patient for the study and induction of general anaesthesia according to local standards and confirmation by the treating anaesthesiologist, that the patient was in stable condition and muscle relaxation was sufficiently reversed by application of 16 mg/kg Sugammadex^®^ (using Train-of-Four with 4/4 stimulus responses and a ratio of > 95%), the phrenic nerve stimulation was initiated. Details of the preparation and anaesthesia induction can be found in Additional file 1.

After changing the MV mode to continuous positive airway pressure (CPAP) with positive end-expiratory pressure (PEEP) set to zero, the STIMIT-exclusive *PMR35 dual coils* in combination with a standard clinical magnetic stimulator were used. The stimulator with a maximal output of 2400 Volt, 160 Joule, pulse length 160 µs at 100% intensity was limited to 50% intensity, i.e. each single coil had a maximal output of 0.55 Tesla and 1200 Volt. The coils were positioned bilaterally on the patient’s neck and phrenic nerve stimulations were attempted with 20% intensity (see Additional file 1: Figure E1). The initial stimulation position (called *capture position*, CP) was identified by varying the coil position on the neck surface and changing the coil angle maintaining the same position. The CP was established when a tidal volume of at least 3 ml/kg ideal body weight was obtained; the time to reach an adequate CP and the tidal volume were documented.

The CP points were marked on the patient’s neck, to identify how the anatomical landmark found through ultrasound differed from the best stimulation point found through electromagnetic stimulation. The skin marking represented the projection of the phrenic nerve itself on the skin, localized through ultrasound in the deep cervical fascia, between the sternocleidomastoid muscle and anterior scalene muscle.

Three series of 10 electromagnetic phrenic nerve stimulations were performed at the specified magnetic field intensity (20%, 30%, 40%, respectively) using *PowerMAG*^*®*^* 100 clinical* stimulator and the STIMIT-exclusive *PMR35* dual coils (both Mag & More GmbH, Germany); between the consequential stimulation series, the patient was ventilated with the same setting as after intubation, followed by a CP search before the start of the following stimulation series. We limited the intensity to 40% intensity in this first application in patients for safety reasons, knowing from self-application and company reports in awake subjects that stimulation at 50% intensity and higher was more likely to be subjectively perceived as uncomfortable or to co-stimulate the brachial plexus. Three additional series of three phrenic nerve stimulations were performed at different magnetic field intensities (10%, 20%, 30%, respectively) and repeated twice to establish a linear relationship assuming that 10% will be insufficient for stimulation. All stimulations were performed as a 2-s-long linear train (without ramp) with 25-Hz frequency. The stimulation interval was manually regulated: as the flow curve reached the baseline (zero line) after a stimulation, the next stimulation was performed. Tidal volume (ml), time to reach the CP between the series and the presence or absence of sternocleidomastoid muscle or arm (plexus brachialis) co-stimulations were documented.

Further stimulations were performed after changing the coil position from the initial CP, to measure the stimulated tidal volume changes with increasing distance from the phrenic nerve: a total of nine stimulations were performed at 5 mm, 10 mm and 15 mm distance from the initial stimulation point moving from medial to lateral (three stimulations for each distance, moving the coils from medial to lateral) using 20% intensity. An additional nine stimulations were performed at 5 mm, 10 mm and 15 mm distance from the initial stimulation point moving from caudal to cranial using 20% intensity. The stimulated tidal volume was documented each time.

### Primary and secondary endpoints

For proof of concept that diaphragmatic pacing with bilateral NEPNS is possible, we used a tidal volume of at least 3 ml/kg ideal body weight (IBW) as a surrogate for adequate diaphragmatic contraction, as this threshold is used clinically in ultra-lung-protective MV. Secondary feasibility endpoints were (1) distance between anatomical landmarks and CP, hypothesizing that optimal CP would coincide with the ultrasound determined position of the phrenic nerve; (2) time to find CP as an important feasibility endpoint for clinical acceptance of the technique; (3) tidal volume change with increasing distance to the initial CP to assess the stability of the stimulation and again feasibility of clinical use of NEPNS. To describe NEPNS from a ventilation mechanics point of view, (4) maximal inspiratory flow, (5) airway pressure, (6) latency between stimulation and inspiration as well as (7) maximal abdominal extension measured with abdominal belts (Sonata Carrying Strap 930260-1-2 equipped with Sonata Pressure Sensor 930393; Löwenstein Medical SE & Co. KG, Germany) during NEPNS was assessed. (8) The variation of the stimulated tidal volumes should inform if the variance increases with higher intensities. (9) For safety, incidents during application and adverse events (if any) occurring from stimulation until 24 h after the procedure were documented.

### Statistical analysis and data depiction

As this was a proof-of-concept study and first application of non-invasive electromagnetic stimulation as a potential therapeutic option in anaesthetized patients, no sample size was calculated. Descriptive statistical analysis was performed using mean and standard deviation. A general linear model was used with repeated measurements and intensity as within-subject variable. Post hoc analysis without *p*-value adjustment was performed comparing the different intensities if the main factor intensity was significant. A *p*-value of 0.05 was used as significant. Statistical analysis was performed using IBM SPSS Statistics 27.0 (SPSS, Chicago, IL), figures were created with the same software or R Version 4.1.1 (R Foundation of Statistical Computing, Vienna, Austria).

## Results

**Fig. 1:**
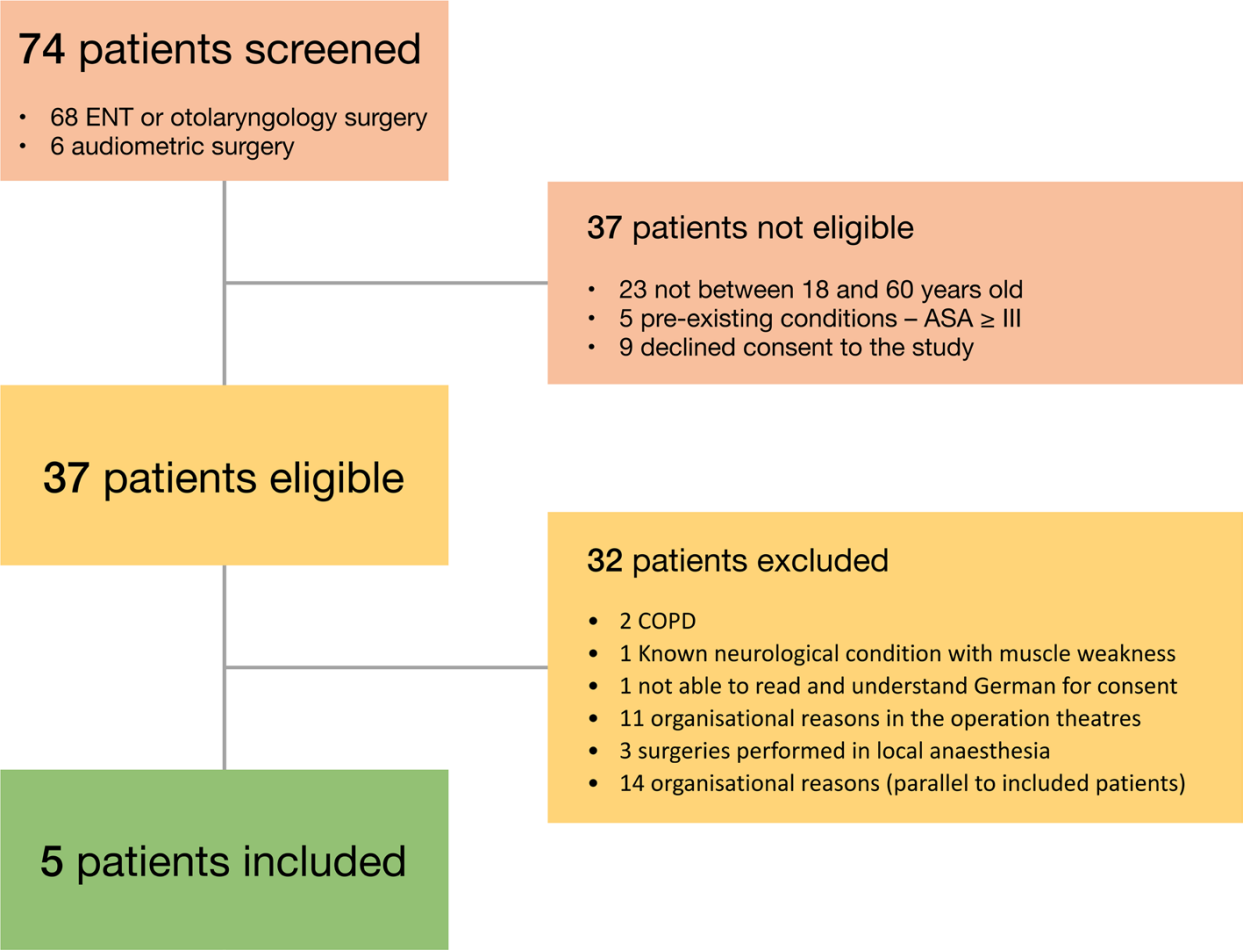
Study flowchart

In total, 74 patients were screened for the study in July 2021. 37 patients were not eligible failing the inclusion criteria and 32 had one or more exclusion criteria. A detailed, summarized diagram of the screening process is shown in Fig. 1. The final population yielded 5 patients; the demographics are shown in Table 1.

**Table 1:** Baseline characteristics

Characteristics	Values	
	Mean ± SD or *n* (%)	Range
Age (years)	27.6 ± 6.7	19–36
Female (*N*, %)	3 (60%)	
Weight (kg)	63.0 ± 5.2	56–69
Height (cm)	168.4 ± 7.2	160–172
Body mass index (kg/m^2^)	22.6 ± 1.14	21–24
Charlson Comorbidity Index	0	0–0
Sugammadex^®^ dosage (mg)	1080 ± 110	

*SD* standard deviation

### Primary endpoint

**Fig. 2:**
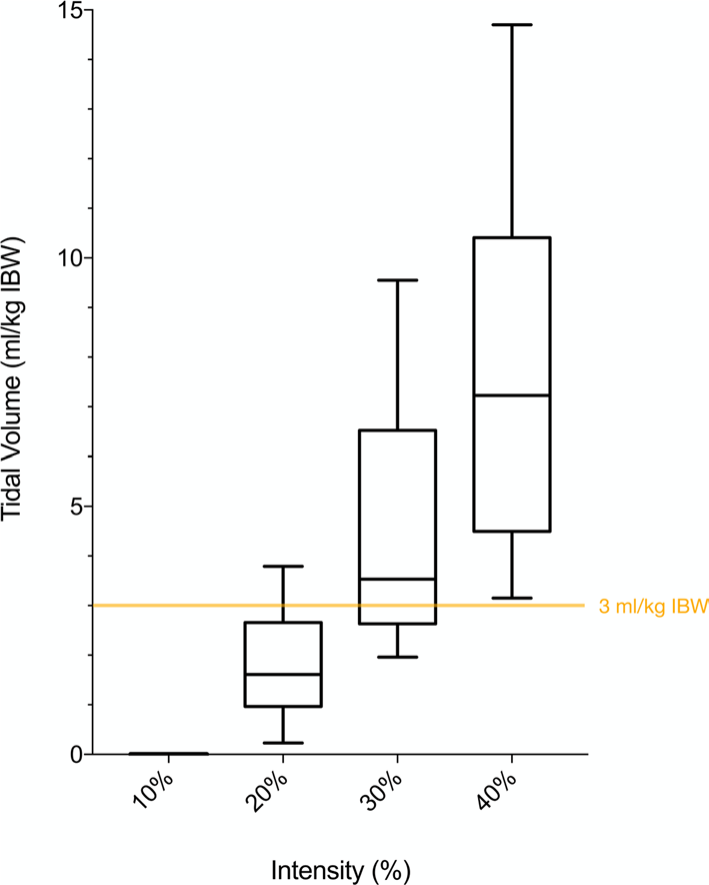
Tidal volumes in ml/kg of ideal body weight [IBW] generated by bilateral non-invasive electromagnetic phrenic nerve stimulation. The boxplot results from individual measurements

**Fig. 3:**
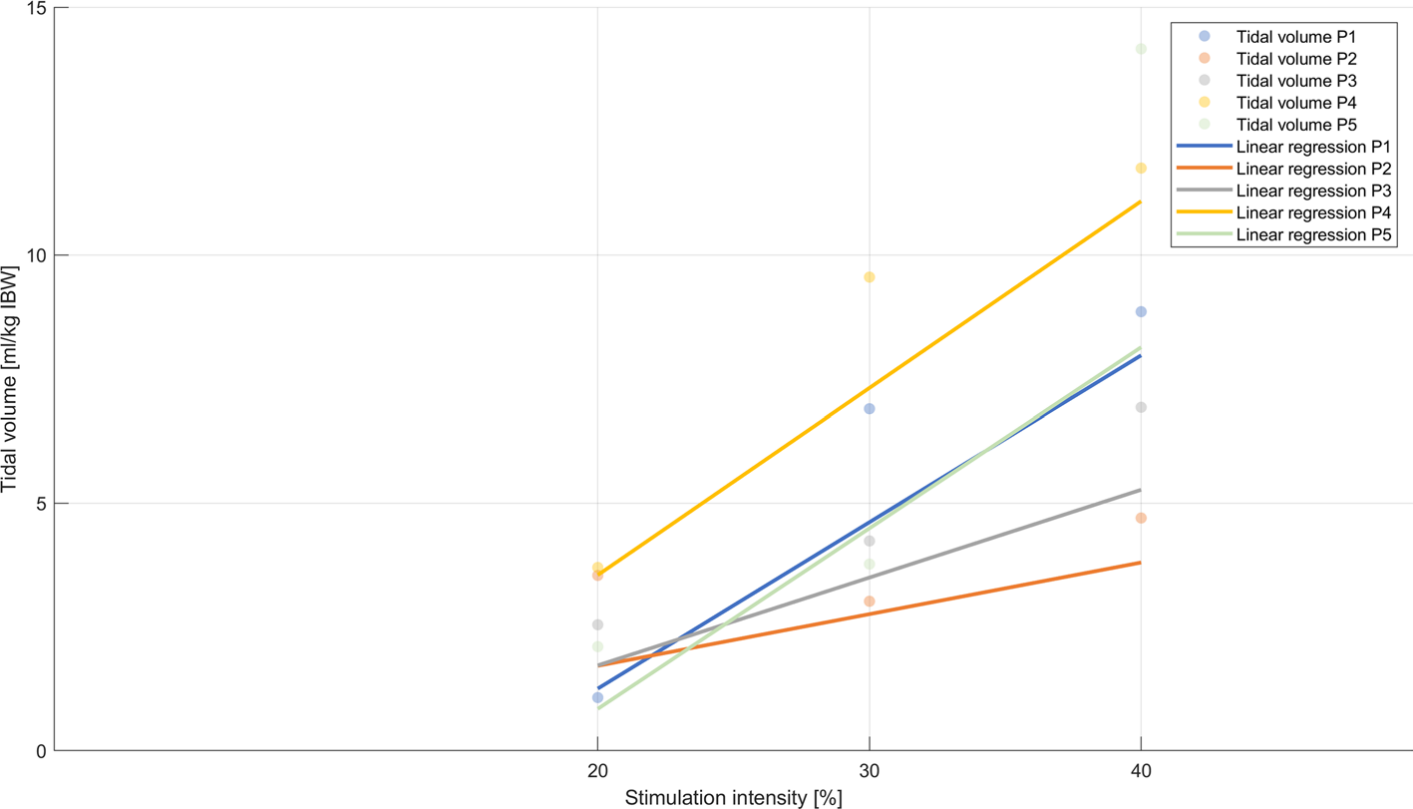
Individual tidal volumes (ml/kg of ideal body weight [IBW]) generated by bilateral non-invasive electromagnetic phrenic nerve stimulation for each patient. Individual measurements are presented as medians of repeated measurements for each patient

All stimulations performed at 40% intensity surpassed the 3 ml/kg IBW threshold set as hypothesis of the present proof-of-concept study. The generated tidal volumes per kilogramme of ideal body weight (IBW) were 0.00 ± 0.00 ml/kg IBW, 1.81 ± 0.99 ml/kg IBW, 4.55 ± 2.23 ml/kg IBW and 7.43 ± 3.06 ml/kg IBW at 10%, 20%, 30% and 40% intensity, respectively (Fig. 2) with a linear relationship between dose (stimulation intensity) and effect (tidal volume) (*p* < 0.001, see Additional file 1: Figure E2). Intra-individual results are shown in Fig. 3, demonstrating the increase in tidal volume with increasing intensity for each patient. Data with actual body weight are presented in the Additional file 1: Figure E3.

### Secondary endpoints

The average distance between the two coils at the initial stimulation point was 12 ± 5 mm on the antero-lateral neck surface. In comparison with the anatomical landmark found with ultrasound assessment, the best stimulation point found with empirical effective coil stimulation was distanced 10 ± 3 mm for the left side and 8 ± 3 mm for the right side. The average time to empirically find the CP was 89 ± 92 s (range 15–441 s) using 20% intensity.

**Fig. 4:**
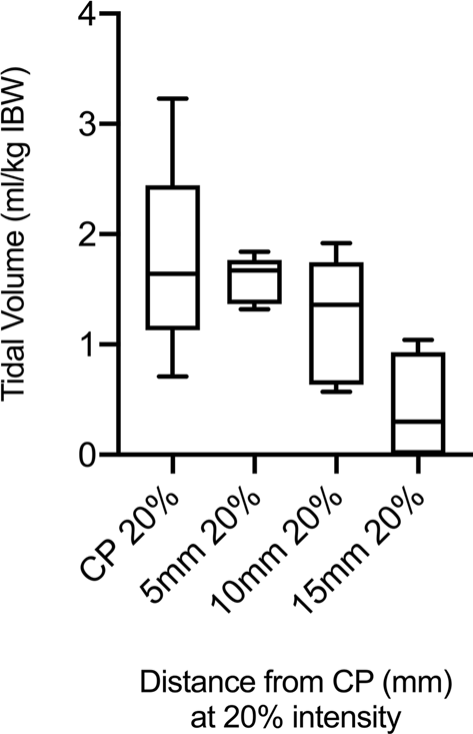
Box plots of tidal volumes generated by bilateral non-invasive electromagnetic phrenic nerve stimulation at 20% intensity by changing coil position by 5 mm, 10 mm and 15 mm from capture point (CP), respectively

Tidal volume reduced gradually as the stimulation was performed at an increasing distance from the optimal stimulation point. The medio-lateral displacement was feasible in all patients. Tidal volume decreased linearly (*p* < 0.001, Fig. 4) as the distance from the used stimulation point was increased anterior to posterior. Mean tidal volumes were 1.58 ± 0.27 ml/kg IBW, 1.25 ± 0.57 ml/kg IBW and 0.73 ± 0.34 ml/kg IBW for 5 mm, 10 mm, and 15 mm distance, respectively.

Due to the anatomical structure of the clavicle and mandible bones, manoeuvring the coils cranially or caudally was only feasible in two patients and limited to a maximum of 10 mm. The resulting mean tidal volume decreased 1.62 ± 0.92 ml/kg IBW (*n* = 6 values; 3 measurements per patient) and 1.32 ± 0.88 ml/kg IBW (*n* = 6 values; 3 measurements per patient) for 5 mm and 10 mm distance, respectively. Due to the lack of feasibility of cranial or caudal coil replacement for most patients, a linear regression for the caudal–cranial positioning change was not performed.

The stimulation was performed with the mechanical ventilator in a spontaneous breathing mode (CPAP mode with zero PEEP). In these conditions, flow is positive and pressure is negative during inspiration, while flow is negative and pressure is positive during expiration.

**Fig. 5:**
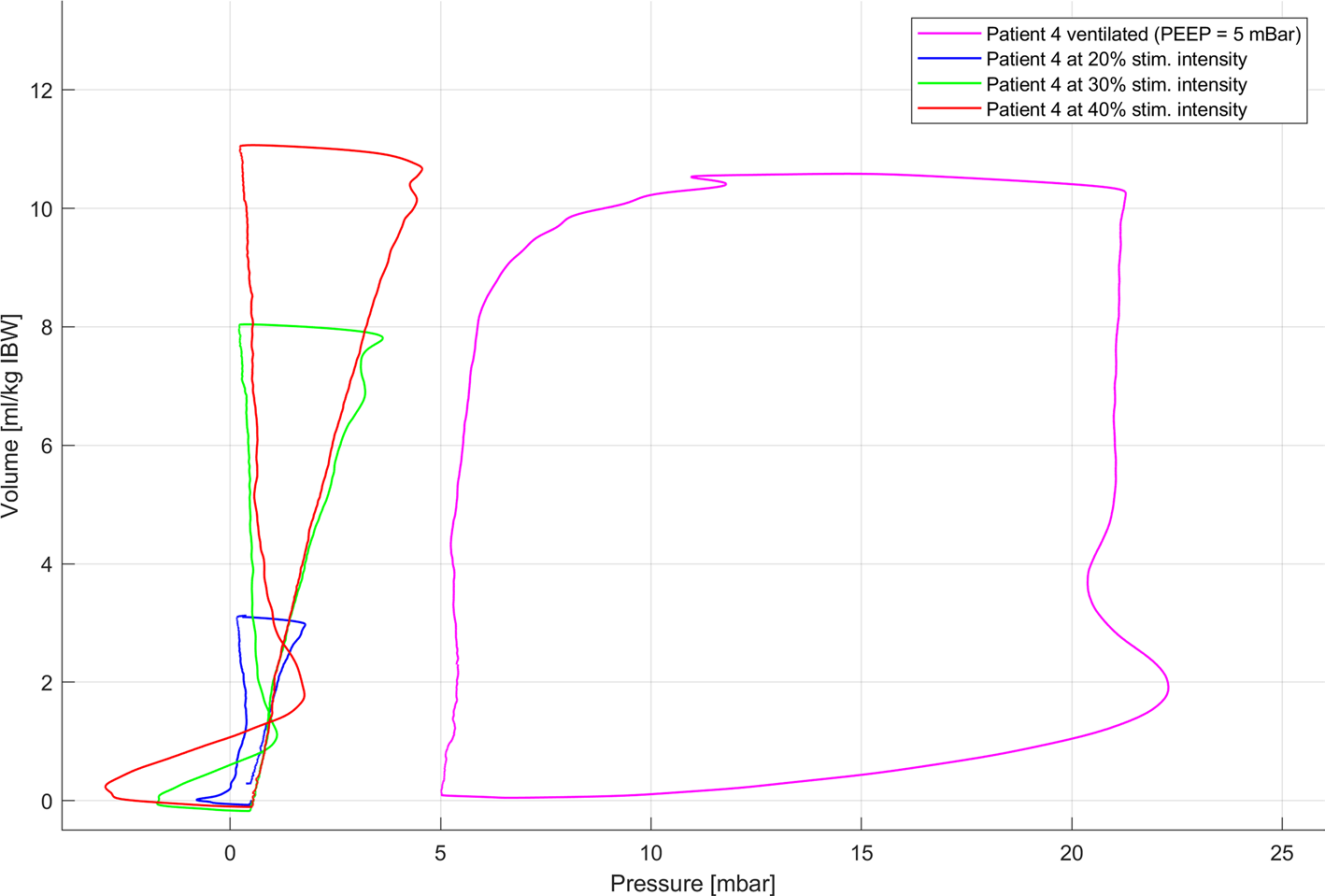
Exemplary pressure–volume loop of one patient. Non-invasive electromagnetic phrenic nerve stimulation was performed with the mechanical ventilator in a spontaneous breathing mode (CPAP mode with zero PEEP). In these conditions, flow was positive and pressure was negative during inspiration, while flow was negative and pressure was positive during expiration (blue, green and red curves with 20%, 30% and 40% stimulation intensity, respectively)

In CPAP mode, peak inspiratory pressure was − 0.5 ± 0.2 cmH_2_O, − 1.3 ± 0.4 cmH_2_O and − 2.7 ± 1.1 cmH_2_O at 20%, 30% and 40% intensity, respectively, while the peak expiratory pressure was 1.9 ± 1.3 cmH_2_O, 2.7 ± 0.6 cmH_2_O and 3.2 ± 1.1 cmH_2_O at 20%, 30% and 40% intensity, respectively. Pressure–volume loops visually showed the expected different pressure–volume loops for MV and stimulated breaths (Fig. 5) with different maximal pressures (see Additional file 1: Figure E4). During MV in passive conditions with 5 cmH_2_O PEEP peak expiratory pressure was 17.2 ± 3.5 cmH_2_O and peak inspiratory pressure 2.3 ± 2.5 cmH_2_O.

**Fig. 6:**
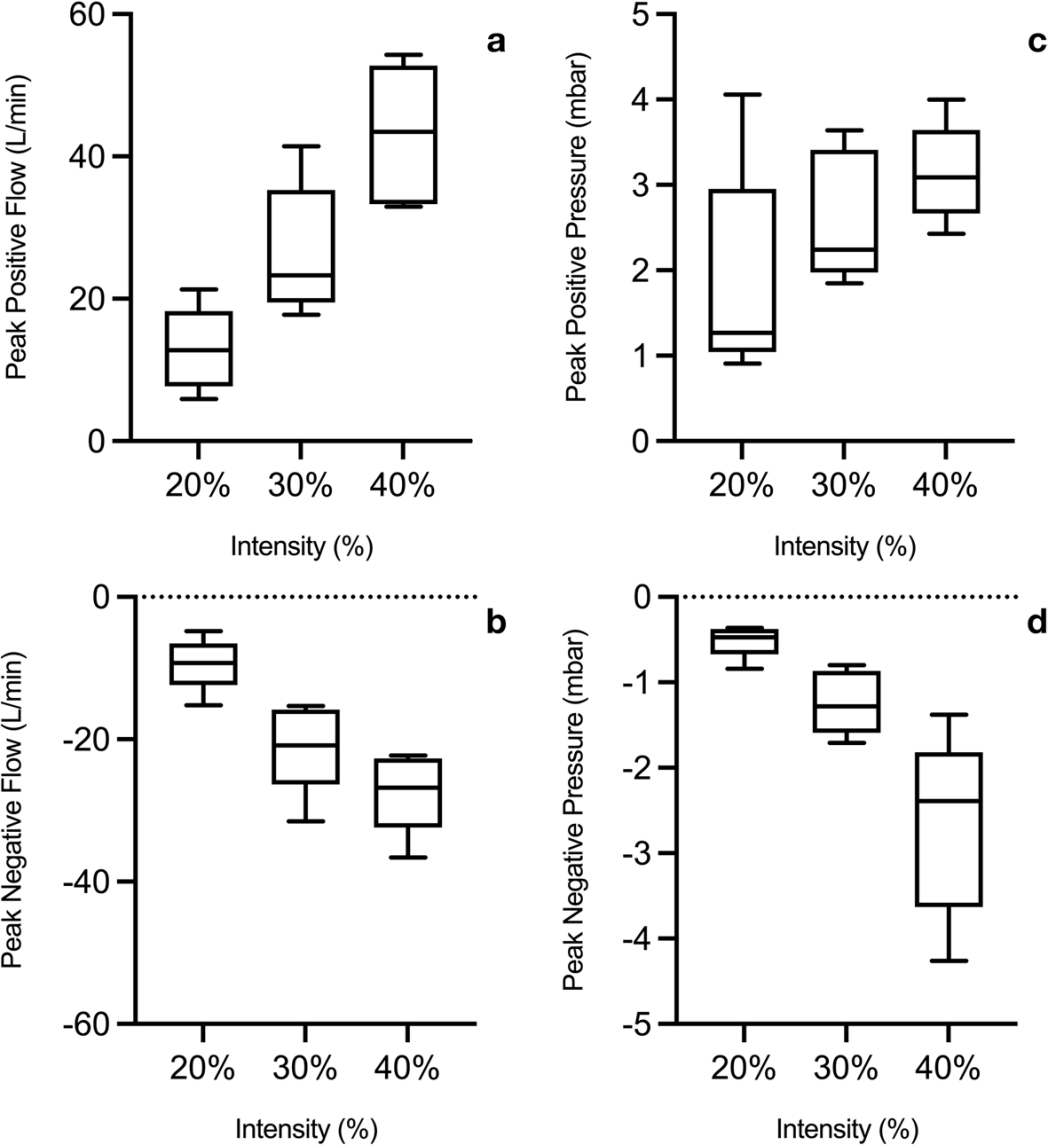
Boxplots presenting positive (**a**, inspiration) and negative (**b**, expiration) flow peaks and positive (**c**, expiration) and negative (**d**, inspiration) pressure peaks at different stimulation intensities during bilateral non-invasive electromagnetic phrenic nerve stimulation. Linear regression in each relationship resulted in a significant effect (*p* < 0.001) for intensity

A linear relationship of both positive (inspiration) and negative (expiration) maximum flow with stimulation intensity was found. The same statistically significant linear proportionality was found for both negative (inspiration) and positive (expiration) maximum pressure (all *p* < 0.001 for intensity, Fig. 6). Single patients’ flow and pressure curves for all intensities are presented in Additional file 1: Figure E4. Averaged tidal volume curves of all patients at different intensities and descriptive statistics are presented in Additional file 1: Figure E5 and Table E1.

The variance over all patients of the stimulated tidal volume generated by NEPNS was 0.97 ml/kg IBW, 4.99 ml/kg IBW and 10.26 ml/kg IBW at 20%, 30% and 40% intensity, respectively (Fig. 3).

The overall contraction latency (all intensities combined) was 26 ± 15 ms. Mean time from stimulation to inspiration (flow ≥ 0.5 L/min) was 28 ± 22 ms, 23 ± 17 ms, and 27 ± 24 ms at 20%, 30% and 40% intensity, respectively, and independent of the stimulation intensity (*p* = 0.68).

The abdominal belt extension measured was 7.08 ± 4.37, 13.41 ± 8.64 and 23.75 ± 11.41 (arbitrary unit) at 20%, 30% and 40% stimulation intensity, respectively. There was a significant effect of intensity (*p* < 0.001) on abdominal extension.

No device incidents occurred. There were two observations: (1) contraction in the upper extremity due to co-stimulation of the plexus brachialis in the majority of cases with stimulation intensities of 30% and beyond, (2) contraction of the sternocleidomastoid muscle due to direct muscle co-stimulation in all cases. The contractions occurred only with stimulation without further consequences for the patient.

Two minor adverse events associated with the stimulation were documented: (1) temporary redness of the skin at the clavicles occurred in 2 out of 5 patients (40%), most likely due to the applied pressure during stimulation. The skin redness was only temporary and disappeared at the follow-up visit 6 h later without further consequences for the patient; (2) one administration of ephedrine not related to the stimulation procedure. None of the patients reported pain related to the procedure.

## Discussion

This is the first in-human study to demonstrate that bilateral NEPNS without volitional component is feasible and can be used to generate a diaphragmatic contractions resulting in a tidal volume above 3 ml/kg IBW in anaesthetized patients without pre-existing pulmonary condition, using coils specifically designed for bilateral peripheral nerve stimulation and powered simultaneously by one stimulator. Thereby, a dose–response relationship was demonstrated, i.e. tidal volumes increased with increasing stimulation intensity.

In our approach, the specially designed dual electromagnetic coils were positioned so that the magnetic field was applied directly to the point where the anatomical landmarks of the phrenic nerve were located. Fine-tuning of the position or angle was feasible with these coils and intubation guaranteed airway control, no leakage and more accurate flow, pressure, and volume measurements, compared with non-invasive ventilation. In previous studies [11, 12] the dimension and shape of the butterfly-shaped TMS coils (70 mm) limited the usability during application on the antero-lateral neck surface to find the stimulation point. A direct comparison between the two hardware settings is not possible, as the combination of stimulator and coils differ substantially.

At 40% stimulation intensity, tidal volumes were always above the 3 ml/kg IBW threshold in the five subjects, which equals ultra-lung protective mechanical ventilation [13], while 20% and 30% did achieve diaphragm contraction, but not our set threshold consistently. At 10% intensity no measurable tidal volume was achieved. This dose–response relationship can be explained by the fact that the magnetic field was applied directly on the skin and propagates in depth by exponential decay in soft tissues: higher intensity is needed to reach deeper anatomical structures [14]. Our achieved tidal volumes were much smaller than Sander et al. had achieved in awake subjects [15]. One explanation could be the different design as outlined above. However, based on the authors’ self-experiments while awake, which also showed significantly higher tidal volumes (no data provided), it can be assumed that volitional components while awake are primarily responsible for this difference.

Importantly, the pressure–volume curves during bilateral NEPNS demonstrated low airway pressures in comparison with MV. This suggests the induction of physiological diaphragmatic breathing, which generates negative pressures during the inspiratory phase and low positive pressures by passive elastic relaxation of the diaphragm during the expiratory phase. Harmful pressures that could lead to barotrauma were avoided with NEPNS [16].

As this technique was used for the first time in patients, feasibility of the stimulation was an important second goal. Time to capture was within a clinically acceptable time frame to find CP, averaging less than 2 min. Furthermore, after finding the CP, the stimulation signal was quite stable, even with 5 mm change the tidal volume was only reduced by below 15%. The latency till a minimal flow achieved was independent by the applied intensity, i.e. the conduction speed through the phrenic nerves seemed to be maintained at the tested intensities, since the impulses travel physiologically always at the same speed in the same subject [17].

Clinical outcomes have not been assessed by our proof-of-concept study. Nevertheless, similar approaches have been used and resulted in improved maximal inspiratory pressure but no difference in MV time (after transvenous diaphragm neurostimulation) [18], increased diaphragm thickness (after percutaneous phrenic nerve stimulation) [19], and shorter MV time in spinal cord injury patients (using transcutaneous electrical diaphragm stimulation) [11, 12]. Bilateral NEPNS can potentially achieve the same positive clinical outcomes without the infectious and thrombotic risks linked to transvenous and percutaneous approaches [20].

As a future perspective, bilateral NEPNS is not meant to substitute physical and respiratory physical therapy [19]. Rescuing diaphragm muscle mass is vital, given that MV leads to atrophy within 72 h after intubation [21]; nevertheless, critically ill patients are often not able to undergo respiratory physical therapy during this time. Bilateral NEPNS has been proposed as a prophylactic therapy to prevent or reduce diaphragm atrophy during the first days after intubation [22] and basal stimulated diaphragm contractions in the spectrum of ultra-lung-protective MV could counteract the pathological mechanisms of VIDD [23], as spontaneous breathing does physiologically.

In terms of safety of the application, we previously assessed the interference of such magnetic field with other medical devices in the ICU setting: at 30 cm distance no interference with other electronic devices should be expected [24]. Magnetic field powered therapies in patients with implanted magnetically sensitive devices (e.g., ICD) should be avoided, even though a multidisciplinary approach could broaden the use for some of these patients [25].

No safety issues were reported in the studies using a similar non-invasive approach [11, 26] and adverse events were not documented. We experienced co-contractions and temporary skin reddening which did not represent a safety risk for the patients; the first is related to the anatomical proximity of the brachial plexus and the latter to the slight pressure of coils on the patient’s skin.

This study has limitations. This is a small proof-of-concept study, i.e. the clinical implications must be confirmed in an adequately powered study. Our findings are limited to anaesthetized, healthy, ASA I–II, patients with normal range BMI. Claims on obese patients or critically ill patients are not possible currently. Previous studies demonstrated that BMI and neck circumference are independent factors for generated diaphragm contraction and tidal volume at different intensities; minute ventilation increased with increasing BMI and decreased with increasing neck circumference [11]. Since there were no previous studies on the amount of diaphragm contraction or phrenic nerve stimulation necessary to achieve an effective VIDD prophylaxis, the endpoint of 3 ml/kg IBW was based on considerations that a tidal volume achieved as in ultra-lung-protective MV would represent an adequate diaphragmatic contraction to counteract VIDD. If this assumption is correct and ameliorates VIDD, has to be evaluated in the future. Nevertheless, the proof of the concept of repeated bilateral NEPNS with subsequent diaphragmatic contraction has been successful.

## Conclusions

Bilateral NEPNS is feasible and can be performed to generate adequate ultra-lung-protective tidal volumes in anaesthetized patients without pre-existing pulmonary condition using new designed coils for bilateral NEPNS. Further studies are needed to assess the feasibility and adequate intensity in critically ill patients. The technique could potentially be used to ventilate deeply sedated patients and could substitute spontaneous diaphragm contraction as a prophylactic or therapeutic strategy to counteract diaphragm atrophy and VIDD.

## Supplementary Information

**Additional file 1.** Appendix with additional methods as well as supplementary figures and tables.

## Data Availability

The datasets generated and/or analysed during the current study are not publicly available due German data protection regulations but are available from the corresponding author on reasonable scientific request through a data share agreement.

## Abbreviations

CI: Confidence interval
CP: Capture point
CPAP: Continuous positive airway pressure
IBW: Ideal body weight
ICU: Intensive care unit
ICUAW: Intensive care unit acquired weakness
MIP: Maximal inspiratory pressure
MV: Mechanical ventilation
NEPNS: Non-invasive electromagnetic phrenic nerve stimulation
OR: Odds ratio
PEEP: Positive end-expiratory pressure
PT: Physical therapy
VIDD: Ventilator-induced diaphragm dysfunction

## References

[1] Berger D, Bloechlinger S, von Haehling S, Doehner W, Takala J, Z'Graggen WJ (2016). Dysfunction of respiratory muscles in critically ill patients on the intensive care unit. J Cachexia Sarcopenia Muscle.

[2] Kilapong B, Aditianingsih D, Sedono R, Sugiarto A, Salamah T (2021). Diaphragm muscle thinning in mechanically ventilated critically ill patients. J Pak Med Assoc.

[3] Levine S, Nguyen T, Taylor N, Friscia ME, Budak MT, Rothenberg P (2008). Rapid disuse atrophy of diaphragm fibers in mechanically ventilated humans. N Engl J Med.

[4] Jung B, Moury PH, Mahul M, de Jong A, Galia F, Prades A (2016). Diaphragmatic dysfunction in patients with ICU-acquired weakness and its impact on extubation failure. Intensive Care Med.

[5] Liu YY, Li LF (2018). Ventilator-induced diaphragm dysfunction in critical illness. Exp Biol Med (Maywood).

[6] Dot I, Pérez-Teran P, Samper M-A, Masclans J-R (2017). Diaphragm dysfunction in mechanically ventilated patients. Archivos de Bronconeumología (English Edition).

[7] Greco M, De Corte T, Ercole A, Antonelli M, Azoulay E, Citerio G (2022). Clinical and organizational factors associated with mortality during the peak of first COVID-19 wave: the global UNITE-COVID study. Intensive Care Med.

[8] Wollersheim T, Grunow JJ, Carbon NM, Haas K, Malleike J, Ramme SF (2019). Muscle wasting and function after muscle activation and early protocol-based physiotherapy: an explorative trial. J Cachexia Sarcopenia Muscle.

[9] Le Bourdelles G, Viires N, Boczkowski J, Seta N, Pavlovic D, Aubier M (1994). Effects of mechanical ventilation on diaphragmatic contractile properties in rats. Am J Respir Crit Care Med.

[10] Jubran A (2006). Critical illness and mechanical ventilation: effects on the diaphragm. Respir Care.

[11] Sander BH, Dieck T, Homrighausen F, Tschan CA, Steffens J, Raymondos K (2010). Electromagnetic ventilation: first evaluation of a new method for artificial ventilation in humans. Muscle Nerve.

[12] Adler D, Gottfried SB, Bautin N, Mirkovic T, Schmidt M, Raux M (2011). Repetitive magnetic stimulation of the phrenic nerves for diaphragm conditioning: a normative study of feasibility and optimal settings. Appl Physiol Nutr Metab.

[13] Abrams D, Agerstrand C, Beitler JR, Karagiannidis C, Madahar P, Yip NH (2022). Risks and benefits of ultra-lung-protective invasive mechanical ventilation strategies with a focus on extracorporeal support. Am J Respir Crit Care Med.

[14] Gomez LJ, Goetz SM, Peterchev AV (2018). Design of transcranial magnetic stimulation coils with optimal trade-off between depth, focality, and energy. J Neural Eng.

[15] Sander BH, Dieck T, Homrighausen F, Tschan CA, Steffens J, Raymondos K. Electromagnetic ventilation: first evaluation of a new method for artificial ventilation in humans. United States2010 2010–9. 305–10 p. 10.1002/mus.21698 20544943

[16] Neto AS, Hemmes SN, Barbas CS, Beiderlinden M, Fernandez-Bustamante A, Futier E (2016). Association between driving pressure and development of postoperative pulmonary complications in patients undergoing mechanical ventilation for general anaesthesia: a meta-analysis of individual patient data. Lancet Respir Med.

[17] Chen R, Collins S, Remtulla H, Parkes A, Bolton CF (1995). Phrenic nerve conduction study in normal subjects. Muscle Nerve.

[18] Dres M, Gama de Abreu M, Merdji H, Müller-Redetzky H, Dellweg D, Randerath WJ, et al. Randomised clinical study of temporary transvenous phrenic nerve stimulation in difficult-to-wean patients. Am J Respir Crit Care Med. 2022. 10.1164/rccm.202107-1709OC PMC9872796 35108175

[19] Soták M, Roubík K, Henlín T, Tyll T (2021). Phrenic nerve stimulation prevents diaphragm atrophy in patients with respiratory failure on mechanical ventilation. BMC Pulm Med.

[20] Lockwood J, Desai N (2019). Central venous access. Br J Hosp Med (Lond).

[21] Duarte GL, Bethiol AL, Ratti L, Franco G, Moreno R, Tonella RM (2021). Transcutaneous electrical diaphragmatic stimulation reduces the duration of invasive mechanical ventilation in patients with cervical spinal cord injury: retrospective case series. Spinal Cord Ser Cases.

[22] Morris IS, Dres M, Goligher EC. Phrenic nerve stimulation to protect the diaphragm, lung, and brain during mechanical ventilation. Intensive Care Med. 2022. 10.1007/s00134-022-06760-8 35688993

[23] Bao Q, Chen L, Chen X, Li T, Xie C, Zou Z (2022). The effects of external diaphragmatic pacing on diaphragm function and weaning outcomes of critically ill patients with mechanical ventilation: a prospective randomized study. Ann Transl Med.

[24] Kuhn KF, Grunow JJ, Leimer P, Lorenz M, Berger D, Schefold JC (2021). Assessment of magnetic flux density properties of electromagnetic noninvasive phrenic nerve stimulations for environmental safety in an ICU environment. Sci Rep.

[25] Ryan JW, Murray AS, Gilligan PJ, Bisset JM, Nolan C, Doyle A (2020). MRI safety management in patients with cardiac implantable electronic devices: utilizing failure mode and effects analysis for risk optimization. Int J Qual Health Care.

[26] Geddes LA, Mouchawar G, Bourland JD, Nyenhuis J (1991). Inspiration produced by bilateral electromagnetic, cervical phrenic nerve stimulation in man. IEEE Trans Biomed Eng.

