# Supplementary material for: First non-invasive magnetic phrenic nerve and diaphragm stimulation in anaesthetized patients: a proof-of-concept study
Source: Intensive Care Med Exp. 2023 Apr 21;11:20. doi: 10.1186/s40635-023-00506-6 (PMC10118662; doi:10.1186/s40635-023-00506-6)
Supplement: Supplementary file 1 — Additional file 1. Appendix with additional methods as well as supplementary figures and tables. [file 40635_2023_506_MOESM1_ESM.pdf]

Online Data Supplement

First non-invasive magnetic phrenic nerve and diaphragm stimulation in anaesthetized patients – a proof-of-concept study

Authors:

Alessandro Panelli[1], Hermann Georges Bartels[1], Sven Krause[2], Michael André Verfuß[1], Aline Michèle Grimm[1], Niklas Martin Carbon[1], Julius J Grunow[1], Diego Stutzer[2], Thomas Niederhauser[2], Laurent Brochard[3,4], Steffen Weber-Carstens[1], Stefan J Schaller[1,5]

1 Charité – Universitätsmedizin Berlin, corporate member of Freie Universität Berlin and Humboldt-Universität zu Berlin, Department of Anesthesiology and Operative Intensive Care Medicine (CVK, CCM), Berlin, Germany

2 Institute for human centered engineering, Bern University of Applied Sciences, Biel/Bienne, Switzerland

3 Keenan Research Centre for Biomedical Science, Li Ka Shing Knowledge Institute, Unity Health Toronto, Toronto, ON, Canada.

4 Interdepartmental Division of Critical Care, University of Toronto, Toronto, Canada.

5 Technical University of Munich, School of Medicine, Klinikum rechts der Isar, Department of Anesthesiology and Intensive Care, Munich, Germany

Content

Content **2**
Methods – Details on preparation and anaesthesia induction **3**
Figure E1 .... **4**
Figure E2 .... **5**
Figure E3 .... **6**
Figure E4 .... **7**
Figure E5 .... **8**
Table E1 .... **9**

Methods – Details on preparation and anaesthesia induction

Patients who had agreed to participate in the study were prepared for their surgery according to the standard operating procedures (SOPs) of the Department of Anesthesiology and Operative Intensive Care Medicine (CVK, CCM) at Charité – Universitätsmedizin Berlin. After arrival in the operation theatres patients were connected to monitoring devices (measuring blood pressure, heart rate and oxygen saturation) and a venous cannula was placed. In addition to the standard preparation patients were connected to measuring devices for the study intervention (abdominal and thoracic belts with included acceleration sensors for measurement of abdominal and thoracic excursion; additional electrocardiography electrodes connected to a polygraphy device and to a surface-electromyography device).

After the pre-induction anaesthesia checklist (including verification of correct identity, knowledge on the part of the patient about the planned surgery and verification of fasting), induction of narcosis was performed by the study physician according to SOPs. After induction of anaesthesia the effect of Rocuronium was reversed by application of Sugammadex® to assure contractability of skeletal muscles, including the diaphragm. The effectivity was monitored using the Train-of-Four method, with 4/4 stimulus responses and a TOF-Ratio of >95% representing an adequate reversal of muscle relaxation. Ventilation was adjusted by the study physician depending on the individual patient and according to the SOPs. In order to start the study intervention, patients had to be cardiovascular stable, i.e., absence of brady-arrhythmia that afford medical treatment, heart rate had to be between 40 and 150 beats per minute, blood lactate ≤ 4.0 mmol/l, cardiovascular support rate (norepinephrine/epinephrin) ≤ 0.2µg/kg/min, fraction of inspired oxygen < 0.5, PEEP < 10mBar and respiration rate < 20/min.

Figure E1

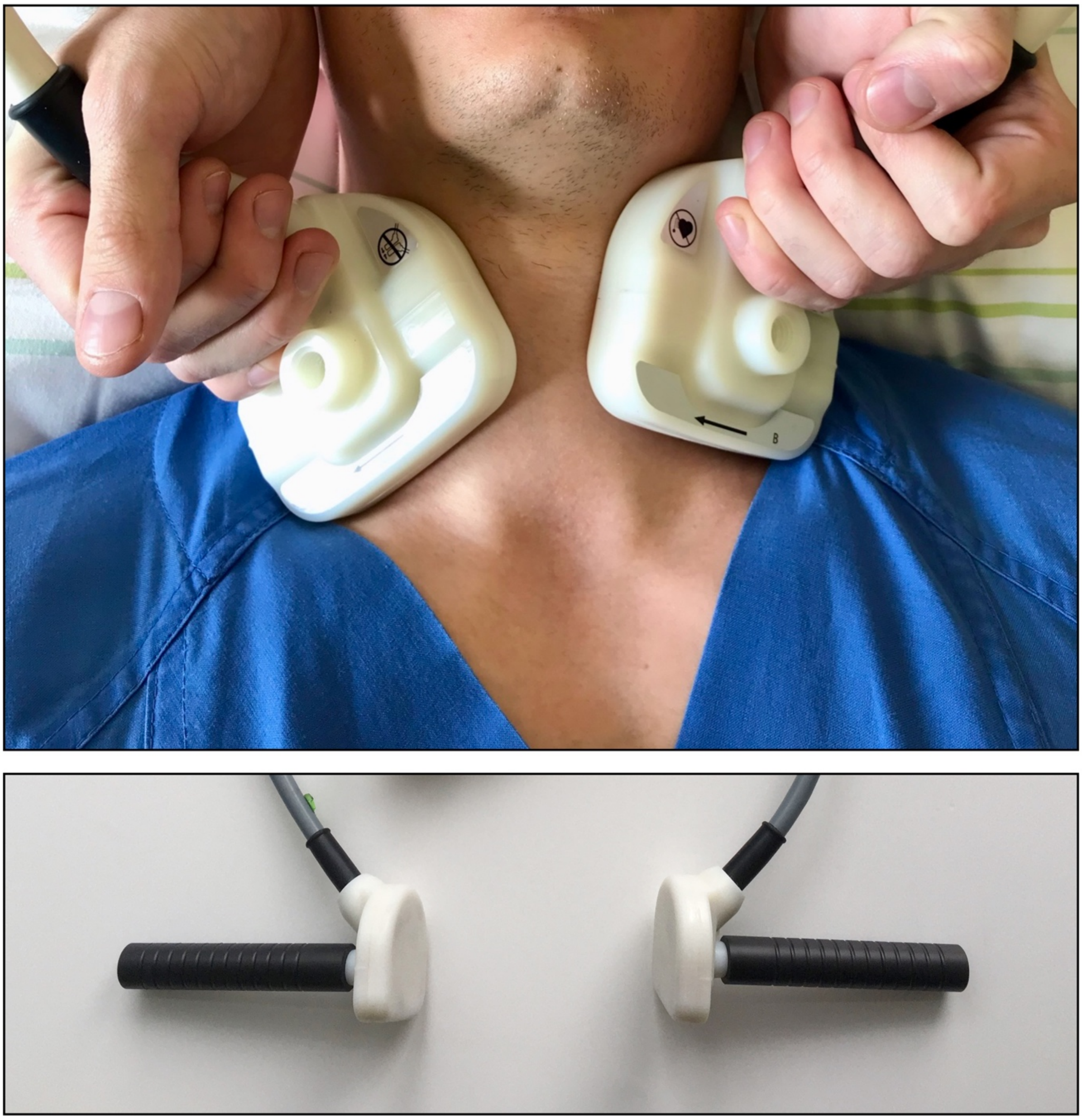

Figure E1. STIMIT-exclusive PMR35 dual coils. Positioning performed on a volunteer (top) in the anterolateral neck surface. Coil shape and handles (bottom).

Figure E2

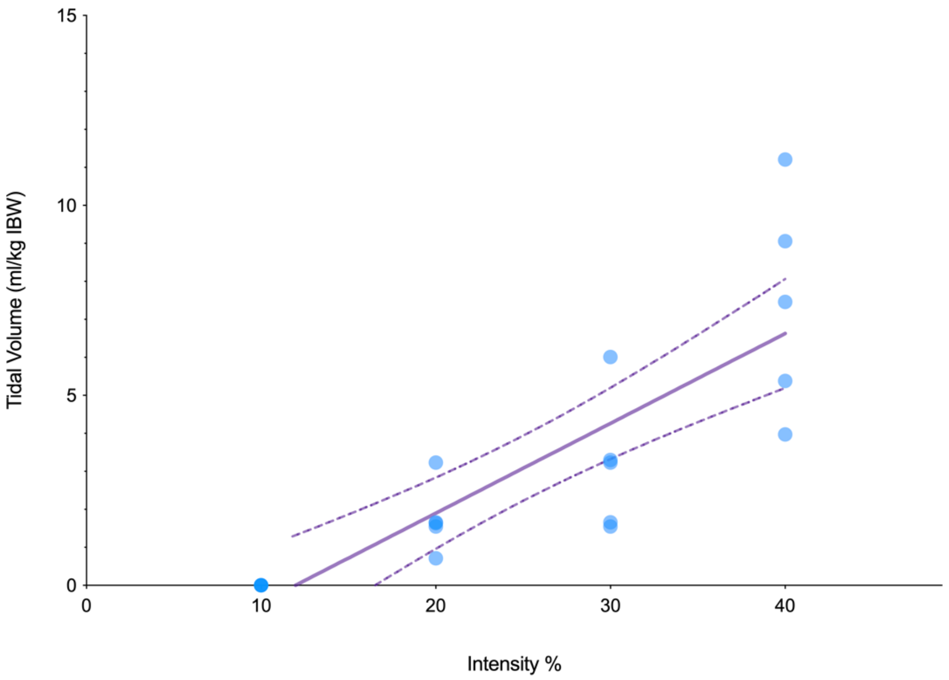

Figure E2. Linear regression for tidal volumes (ml/kg IBW) and stimulation intensity (%). Linear regression with 95 % confidence interval is presented. $p < 0.001$, $r^2 = 0.7$. Each point represents the median value of tidal volumes for each individual patient.

Figure E3

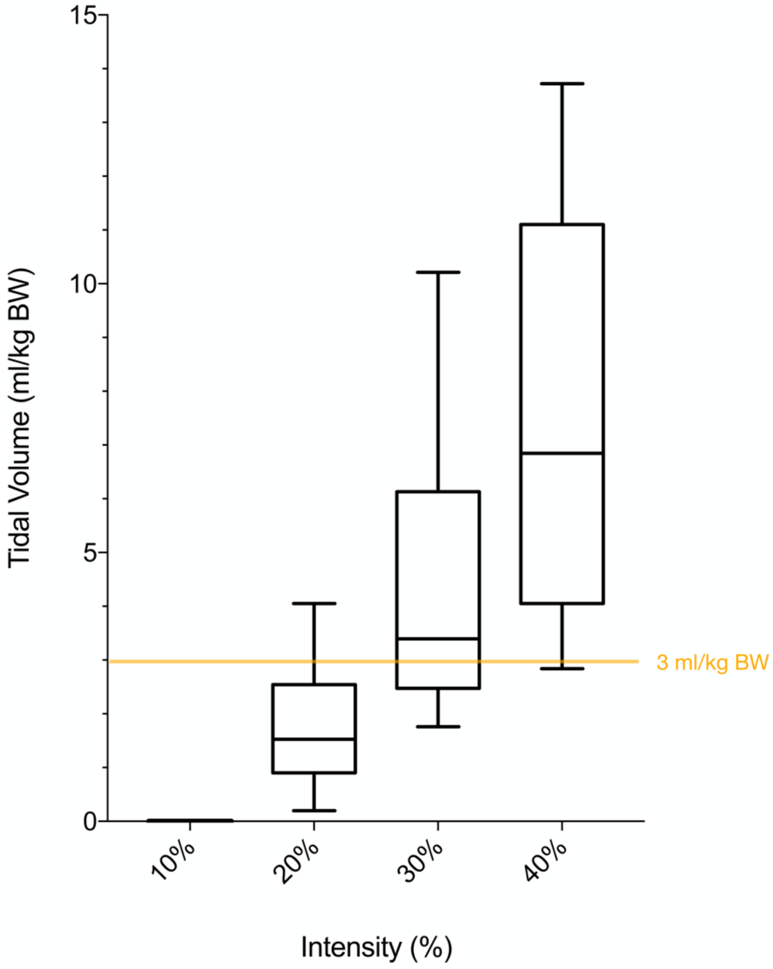

Figure E3. Tidal volumes generated by bilateral non-invasive electromagnetic phrenic nerve stimulation in ml/kg of actual body weight [BW]. The mean generated tidal volumes per kilogram of actual body weight were 0.00 ± 0.00 ml/kg, 1.79 ± 1.07 ml/kg, 4.48 ± 2.47 ml/kg and 7.25 ± 3.27 ml/kg at 10%, 20%, 30%, and 40% intensity, respectively.

Figure E4

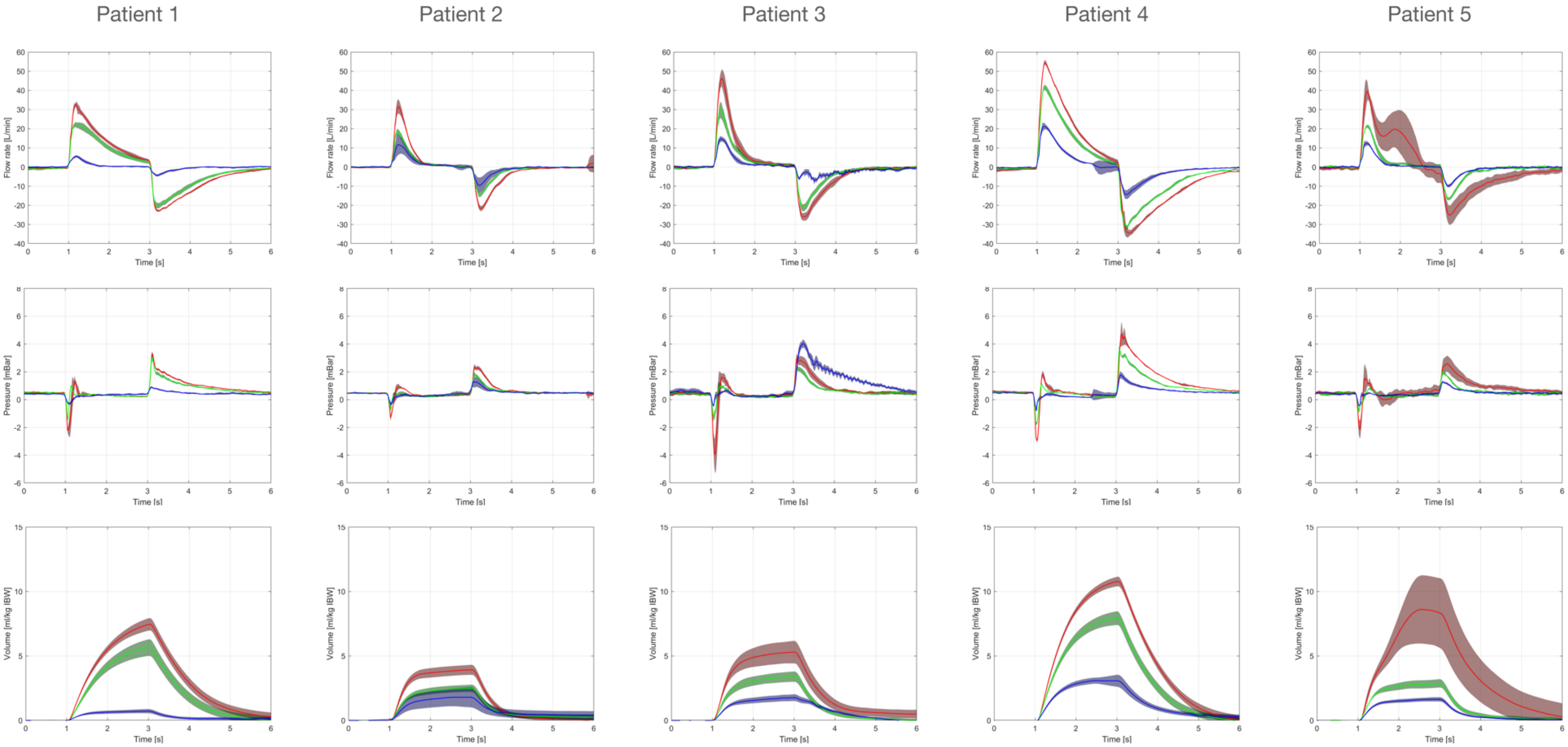

Figure E4. Flow, pressure, and volume curves for all patients at 20% (blue), 30% (green), 40% (red) intensity with 95% confidence intervals.

Figure E5

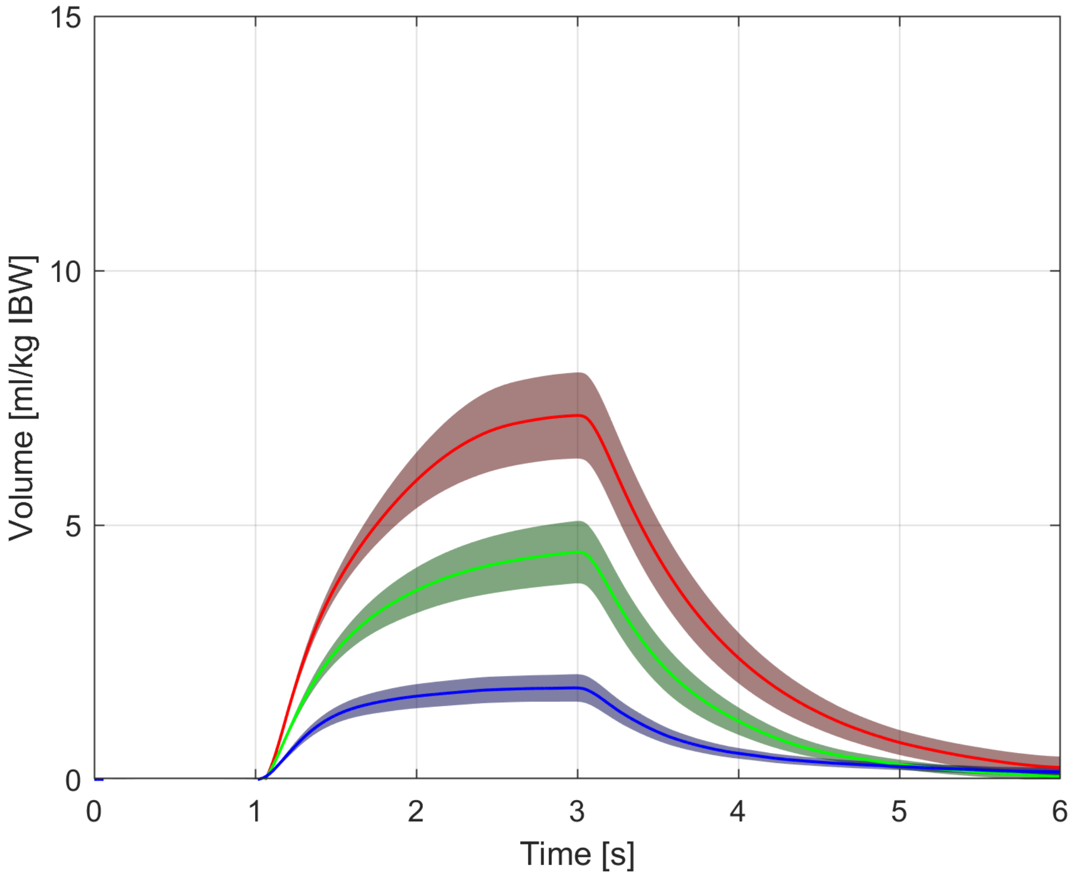

Figure E5. Tidal volumes after bilateral non-invasive electromagnetic phrenic nerve stimulation at 20% (blue), 30% (green) and 40% (red) intensities with 95% confidence intervals for all patients.

Table E1

Table E1. Descriptive statistics of tidal volume (mL/kg IBW) during NEPNS stimulation at 20%, 30% and 40% intensity.

Intensity	n	Minimum	Maximum	Mean	Standard Deviation	Variance (%)
20%	50	0.20	4.05	1.79	1.06	1.13 (63)
30%	50	1.76	10.21	4.48	2.47	6.11 (136)
40%	50	0.23	13.72	7.07	3.40	11.59 (164)
